# Supplementary material for: HIVprotI: an integrated web based platform for prediction and design of HIV proteins inhibitors
Source: J Cheminform. 2018 Mar 9;10:12. doi: 10.1186/s13321-018-0266-y (PMC5845081; doi:10.1186/s13321-018-0266-y)
Supplement: Supplementary file 2 — Additional file 2. Source code of HIVProtI web server. [file 13321_2018_266_MOESM2_ESM.zip › HIVprotI_Source-code/hivprotI/weka-3-6-6/changelogs/CHANGELOG-3-5-0.html]

Weka-3-5-0 Changelog


# Weka-3-5-0 Changelog

Designed for use with Ant.

---

2005-07-04 04:07 mhall

```
Moved the pointer and new buttons
```

- gui/beans/KnowledgeFlowApp.html (1.7)

2005-07-04 03:14 mhall

```
Changed so that the link in the docs now points to the ExplorerGuide
```

- build.xml (1.26)

2005-07-04 03:12 eibe

```
Changed year and URL in LogPanel.
```

- gui/explorer/Explorer.html (1.33)

2005-07-04 00:56 eibe

```
FilteredClassifier now extends SingleClassifierEnhancer. Note that this means that the double hyphen construct now needs to be used to specify the classifier options. Another change: Discretize is now the default filter.
```

- classifiers/meta/FilteredClassifier.html (1.21)

2005-07-04 00:54 eibe

```
Fixed some bugs in handling of option setting errors.
```

- classifiers/SingleClassifierEnhancer.html (1.3)

2005-07-04 00:35 mhall

```
Fixed javadoc
```

- classifiers/meta/AttributeSelectedClassifier.html (1.18)

2005-07-04 00:20 eibe

```
Now extends RandomizableSingleClassifierEnhancer for simplified option handling. Also changed the values of some default parameters so that the classifier is applicable to more datasets with its default options.
```

- classifiers/meta/RacedIncrementalLogitBoost.html (1.5)

2005-07-04 00:04 mhall

```
Now extends the appropriate base class
```

- classifiers/meta/CostSensitiveClassifier.html (1.19)
- classifiers/meta/OrdinalClassClassifier.html (1.12)

2005-07-03 23:59 eibe

```
Decorate now extends RandomizableIteratedSingleClassifierEnhancer for simplified option handling. Unfortunately, the letters for the command line options had to be changed. What used to be I is now E, and what used to be M is now I. Also, the maximum and desired number of iterations are now both set to 10 by default (instead of 50 and 15 respectively).
```

- classifiers/meta/Decorate.html (1.4)

2005-07-03 23:16 eibe

```
Now allows random codes with fewer columns than classes (handy for datasets with lots of classes). Now implements RandomizableSingleClassifierEnhancer for easier option handling.
```

- classifiers/meta/MultiClassClassifier.html (1.39)

2005-07-03 22:55 eibe

```
Changed letter for command line option speciyfing tolerance parameter as it conflicted with the option for specifying a test set.
```

- classifiers/functions/SMO.html (1.55)

2005-07-03 22:50 eibe

```
Now implements RandomizableSingleClassifierEnhancer to simplify option handling.
```

- classifiers/meta/ThresholdSelector.html (1.32)

2005-07-01 01:57 mhall

```
Fixed bug in ungrouping for MetaBeans
```

- gui/beans/BeanInstance.html (1.5)
- gui/beans/MetaBean.html (1.3)

2005-06-30 21:28 mhall

```
Fixed bug in option description
```

- filters/unsupervised/attribute/MathExpression.html (1.2)

2005-06-30 05:42 mhall

```
branches:  1.1.2;
Initial import
```

- core/version.txt (1.1)

2005-06-30 05:41 mhall

```
Initial import
```

- gui/beans/icons/New24.gif (1.1)

2005-06-30 05:27 fracpete

```
fixed getOptions: confid. level (-C) and number of folds (-N) depend differently on reduced error pruning
```

- classifiers/rules/PART.html (1.3)

2005-06-30 04:51 fracpete

```
setOptions: -L instead of -M for MaxBoostingIterations used
```

- classifiers/functions/SimpleLogistic.html (1.6)

2005-06-30 04:29 fracpete

```
getOptions fixed: -S removed (leftover?) and -B only is returned if there's a BIFFile
```

- classifiers/bayes/BayesNet.html (1.23)

2005-06-30 04:09 fracpete

```
getOptions fixed: -F and value are now returned in two strings
```

- classifiers/bayes/AODE.html (1.10)

2005-06-30 03:07 mhall

```
Now reads version number from weka/core/version.txt. This allows the ant release target to automatically set the version number for a release
```

- core/Version.html (1.4)

2005-06-29 22:52 mhall

```
Fixed a bug in NormalizedPolyKernel that resulted in an attempted access to the cache for test instances
```

- classifiers/functions/supportVector/CachedKernel.html (1.2)
- classifiers/functions/supportVector/NormalizedPolyKernel.html (1.2)

2005-06-28 23:50 mhall

```
Updated
```

- gui/beans/README\_KnowledgeFlow (1.5)

2005-06-28 04:25 mhall

```
Some semi-major changes to allow for components to be grouped together
```

- gui/beans/AbstractDataSink.html (1.5)
- gui/beans/AbstractEvaluator.html (1.3)
- gui/beans/AbstractTestSetProducer.html (1.3)
- gui/beans/AbstractTrainAndTestSetProducer.html (1.3)
- gui/beans/AbstractTrainingSetProducer.html (1.3)
- gui/beans/BeanCommon.html (1.2)
- gui/beans/BeanConnection.html (1.3)
- gui/beans/BeanVisual.html (1.5)
- gui/beans/ClassAssigner.html (1.10)
- gui/beans/ClassValuePicker.html (1.4)
- gui/beans/Classifier.html (1.19)
- gui/beans/Filter.html (1.13)
- gui/beans/ModelPerformanceChart.html (1.5)
- gui/beans/PredictionAppender.html (1.10)
- gui/beans/StripChart.html (1.11)

2005-06-27 23:27 fracpete

```
fixed sorting of numeric columns
missing values now only with grey background, no longer displays ? (e.g., anneal dataaset contains '?' as attribute value)
setting a null value for any cell is now interpreted as missing value
```

- gui/arffviewer/ArffTable.html (1.3)
- gui/arffviewer/ArffTableModel.html (1.3)

2005-06-27 23:25 fracpete

```
added constructors for default directory
```

- gui/arffviewer/FileChooser.html (1.2)

2005-06-27 23:24 fracpete

```
moved most stuff from the JFrame to a JPanel - for easier integration in other frames
FileChooser now points to the current directory, no longer to the user's default one
```

- gui/arffviewer/ArffViewer.html (1.6)

2005-06-27 23:24 fracpete

```
branches:  1.1.2;
moved most stuff from the JFrame to a JPanel - for easier integration in other frames
FileChooser now points to the current directory, no longer to the user's default one
```

- gui/arffviewer/ArffViewerMainPanel.html (1.1)

2005-06-27 23:23 mhall

```
Fixed small bug in icon loading
```

- gui/beans/Clusterer.html (1.3)

2005-06-27 23:22 mhall

```
More icons from Jeffrey
```

- gui/beans/icons/DiamondPlain.gif (1.1)
- gui/beans/icons/ModelPerformanceChart.gif (1.1)
- gui/beans/icons/ModelPerformanceChart\_animated.gif (1.1)

2005-06-27 23:22 mhall

```
branches:  1.1.2;
More icons from Jeffrey
```

- gui/beans/icons/Cobweb.gif (1.1)
- gui/beans/icons/Cobweb\_animated.gif (1.1)
- gui/beans/icons/EM.gif (1.1)
- gui/beans/icons/EM\_animated.gif (1.1)
- gui/beans/icons/FarthestFirst.gif (1.1)
- gui/beans/icons/FarthestFirst\_animated.gif (1.1)
- gui/beans/icons/MakeDensityBasedClusterer.gif (1.1)
- gui/beans/icons/MakeDensityBasedClusterer\_animated.gif (1.1)
- gui/beans/icons/SimpleKMeans.gif (1.1)
- gui/beans/icons/SimpleKMeans\_animated.gif (1.1)

2005-06-27 20:33 fracpete

```
fixed save-dialog (JPEG/EPS)
added DISPOSE_ON_CLOSE in main-method to avoid hanging
```

- gui/treevisualizer/TreeVisualizer.html (1.10)

2005-06-27 02:16 fracpete

```
if input/outputcenter file are NULL, they're not returned in getOptions
```

- clusterers/XMeans.html (1.9)

2005-06-22 21:38 eibe

```
Patch from Julien Nioche for faster toString() method.
```

- estimators/DiscreteEstimator.html (1.7)

2005-06-22 21:24 eibe

```
Degrees of freedom can now be set programmatically.
```

- experiment/PairedStats.html (1.8)

2005-06-22 06:46 fracpete

```
restarting of GUI removed, since it created an endless loop, in case the KnowledgeFlow crased with an OutOfMemory Exception
```

- gui/GUIChooser.html (1.23)

2005-06-22 05:58 fracpete

```
TextNodes now turn these characters into entities: <>&'"
otherwise is wasn't possible to add "x < 2" in the notes of an experiment ("<" isn't allowed inside a tag!)
```

- core/xml/XMLSerialization.html (1.6)

2005-06-22 04:51 eibe

```
Now throw exception when there are string attributes. RandomTree had a bug when the class was the 2nd attribute.
```

- classifiers/trees/REPTree.html (1.20)
- classifiers/trees/RandomTree.html (1.11)

2005-06-22 04:23 eibe

```
Fixed spelling mistake.
```

- experiment/RemoteExperimentSubTask.html (1.7)

2005-06-21 03:53 fracpete

```
added -U <username> and -P <password> as commandline options
if query doesn't return a ResultSet like DELETE/UPDATE/INSERT, then a message of how many rows were affected is output to System.err and retrieveInstances(String) returns NULL -  an exception is no longer thrown in that case
```

- experiment/InstanceQuery.html (1.16)

2005-06-20 05:02 fracpete

```
fixed the getOptions() method
```

- core/converters/DatabaseLoader.html (1.3)
- core/converters/DatabaseSaver.html (1.2)

2005-06-19 01:58 fracpete

```
println(Object) now outputs also the name of the Throwable, not just the message
```

- core/Tee.html (1.2)

2005-06-18 02:42 fracpete

```
inserted another System.gc() after the threads are stopped
```

- core/Memory.html (1.2)

2005-06-18 00:33 fracpete

```
databases like Oracle can have datatypes like "DOUBLE PRECISION", but property names cannot contain blanks. blanks in the type name are now always replaced with underscores.
```

- experiment/DatabaseUtils.html (1.20)

2005-06-14 23:17 fracpete

```
columns weren't aligned correctly if a value in the previous column was missing
```

- experiment/ResultMatrixPlainText.html (1.4)

2005-06-14 04:01 fracpete

```
added memory management
```

- gui/sql/SqlViewer.html (1.3)

2005-06-14 03:59 fracpete

```
moved some of the memory management code to weka.core.Memory
error-dialog now contains more information and help
```

- gui/experiment/Experimenter.html (1.10)

2005-06-14 03:04 fracpete

```
frame that replaces System.out/System.err with instances of Tee and outputs the data to the orginal printstreams and the JTextPane in the frame
```

- gui/LogWindow.html (1.1)

2005-06-09 21:21 fracpete

```
fixed tooltiptext for save dialog (ctrl -> shit)
```

- gui/AttributeVisualizationPanel.html (1.21)

2005-06-09 02:36 mhall

```
Fixed a couple of event names
```

- gui/beans/ClassifierPerformanceEvaluatorBeanInfo.html (1.4)

2005-06-08 04:49 mhall

```
branches:  1.1.2;
Initial import
```

- gui/beans/VisualizableErrorEvent.html (1.1)
- gui/beans/VisualizableErrorListener.html (1.1)

2005-06-08 04:47 mhall

```
Made a couple of utility methods public and static
```

- gui/GenericObjectEditor.html (1.42)

2005-06-08 01:37 mhall

```
Can now plot classifier errors
```

- gui/beans/ClassifierPerformanceEvaluator.html (1.11)

2005-06-08 01:35 mhall

```
Removed some unnecessary import statements
```

- attributeSelection/ASEvaluation.html (1.10)
- attributeSelection/AttributeEvaluator.html (1.8)
- attributeSelection/AttributeSelection.html (1.37)
- attributeSelection/AttributeSetEvaluator.html (1.2)
- attributeSelection/AttributeTransformer.html (1.7)
- attributeSelection/CfsSubsetEval.html (1.20)
- attributeSelection/ChiSquaredAttributeEval.html (1.9)
- attributeSelection/ErrorBasedMeritEvaluator.html (1.5)
- attributeSelection/ExhaustiveSearch.html (1.10)
- attributeSelection/FCBFSearch.html (1.2)
- attributeSelection/GainRatioAttributeEval.html (1.17)
- attributeSelection/GreedyStepwise.html (1.5)
- attributeSelection/HoldOutSubsetEvaluator.html (1.4)
- attributeSelection/InfoGainAttributeEval.html (1.15)
- attributeSelection/OneRAttributeEval.html (1.15)
- attributeSelection/PrincipalComponents.html (1.27)
- attributeSelection/RaceSearch.html (1.16)
- attributeSelection/RandomSearch.html (1.13)
- attributeSelection/RankSearch.html (1.12)
- attributeSelection/RankedOutputSearch.html (1.10)
- attributeSelection/Ranker.html (1.22)
- attributeSelection/ReliefFAttributeEval.html (1.16)
- attributeSelection/SVMAttributeEval.html (1.18)
- attributeSelection/StartSetHandler.html (1.7)
- attributeSelection/SubsetEvaluator.html (1.8)
- attributeSelection/SymmetricalUncertAttributeEval.html (1.16)
- attributeSelection/SymmetricalUncertAttributeSetEval.html (1.2)
- attributeSelection/UnsupervisedAttributeEvaluator.html (1.7)
- attributeSelection/UnsupervisedSubsetEvaluator.html (1.7)
- attributeSelection/WrapperSubsetEval.html (1.24)

2005-06-05 10:43 fracpete

```
open DB button now pops up the SqlViewerDialog
```

- gui/explorer/PreprocessPanel.html (1.53)

2005-06-05 10:35 fracpete

```
fixed minor display bugs
```

- gui/sql/ResultSetTableModel.html (1.2)

2005-06-05 02:25 fracpete

```
modified copyright
```

- experiment/xml/XMLExperiment.html (1.2)

2005-06-05 02:23 fracpete

```
branches:  1.1.2;
images for the sqlviewer
```

- gui/images/empty\_small.gif (1.1)
- gui/images/error\_small.gif (1.1)
- gui/images/information\_small.gif (1.1)
- gui/images/question\_small.gif (1.1)

2005-06-05 02:22 fracpete

```
little SQL worksheet for querying databases
```

- gui/sql/event/ConnectionEvent.html (1.1)
- gui/sql/event/ConnectionListener.html (1.1)
- gui/sql/event/HistoryChangedEvent.html (1.1)
- gui/sql/event/HistoryChangedListener.html (1.1)
- gui/sql/event/QueryExecuteEvent.html (1.1)
- gui/sql/event/QueryExecuteListener.html (1.1)
- gui/sql/event/ResultChangedEvent.html (1.1)
- gui/sql/event/ResultChangedListener.html (1.1)

2005-06-05 02:21 fracpete

```
little SQL worksheet for querying databases
```

- gui/sql/ConnectionPanel.html (1.1)
- gui/sql/DbUtils.html (1.1)
- gui/sql/InfoPanel.html (1.1)
- gui/sql/InfoPanelCellRenderer.html (1.1)
- gui/sql/QueryPanel.html (1.1)
- gui/sql/ResultPanel.html (1.1)
- gui/sql/ResultSetHelper.html (1.1)
- gui/sql/ResultSetTable.html (1.1)
- gui/sql/ResultSetTableCellRenderer.html (1.1)
- gui/sql/SqlViewerDialog.html (1.1)

2005-06-05 02:16 fracpete

```
modified copyright and added filter name removal for summary, key, etc.
```

- experiment/ResultMatrixCSV.html (1.2)
- experiment/ResultMatrixGnuPlot.html (1.3)
- experiment/ResultMatrixHTML.html (1.2)
- experiment/ResultMatrixLatex.html (1.3)
- experiment/ResultMatrixSignificance.html (1.2)

2005-06-05 02:14 fracpete

```
modified copyright and extended removal of filter names (didn't work correctly if rows/cols transposed in experimenter)
```

- experiment/ResultMatrix.html (1.5)

2005-06-05 02:12 fracpete

```
modified copyright and added support for several classes: hashmap, hashset, linkedlist, properties, stack, treemap, treeset, vector
```

- core/xml/XMLBasicSerialization.html (1.2)

2005-06-05 02:12 fracpete

```
modified copyright
```

- experiment/Tester.html (1.3)

2005-06-05 02:10 fracpete

```
modified copyright
```

- core/xml/KOML.html (1.2)
- core/xml/MethodHandler.html (1.2)
- core/xml/PropertyHandler.html (1.2)
- core/xml/SerialUIDChanger.html (1.2)
- core/xml/XMLDocument.html (1.2)
- core/xml/XMLOptions.html (1.2)
- core/xml/XMLSerializationMethodHandler.html (1.2)

2005-06-05 02:09 fracpete

```
modified copyright
```

- core/InstanceComparator.html (1.2)

2005-06-05 02:07 fracpete

```
modified copyright
```

- classifiers/xml/XMLClassifier.html (1.4)

2005-06-03 02:45 mhall

```
Changed to enable incremental loading to be restarted on the existing source
```

- core/converters/AbstractLoader.html (1.7)
- core/converters/ArffLoader.html (1.10)
- core/converters/C45Loader.html (1.10)
- core/converters/Loader.html (1.7)

2005-06-03 02:45 mhall

```
Fixed so that incremental loading can be restarted on the same source
```

- gui/beans/Loader.html (1.12)

2005-05-31 04:51 mhall

```
Modified to accomodate the code shift from KnowledgeFlow to KnowledgeFlowApp
```

- gui/beans/Saver.html (1.2)

2005-05-31 04:35 mhall

```
branches:  1.1.2;
Listener for something that wants to be notified when something else has completed starting up (e.g. the KnowledgeFlow).
```

- gui/beans/StartUpListener.html (1.1)

2005-05-31 04:33 mhall

```
branches:  1.1.2;
Initial import
```

- gui/beans/icons/splash.jpg (1.1)

2005-05-31 04:31 mhall

```
Code moved to KnowledgeFlowApp. Now displays a splash screen and launches the KnowledgeFlowApp
```

- gui/beans/KnowledgeFlow.html (1.28)

2005-05-31 04:29 mhall

```
branches:  1.1.2;
Initial import
```

- gui/SplashWindow.html (1.1)

2005-05-26 04:17 eibe

```
Fixed bug in documentation.
```

- classifiers/meta/CVParameterSelection.html (1.29)

2005-05-24 22:40 mhall

```
Fixed bug relating to the use of ReplaceMissingValues filter when a class attribute is set in the data
```

- clusterers/EM.html (1.33)
- clusterers/SimpleKMeans.html (1.23)

2005-05-17 02:59 mhall

```
HashKey can now treat the class as a normal attribute if required
```

- classifiers/rules/DecisionTable.html (1.30)

2005-05-14 06:05 fracpete

```
updated classes to reflect changes in Tester/ResultMatrix
```

- gui/experiment/OutputFormatDialog.html (1.3)
- gui/experiment/ResultsPanel.html (1.35)

2005-05-14 06:00 fracpete

```
removed methods that are no longer used
moved mean/stddev precisions into ResultMatrix
ResultMatrix is now an instance and no longer a class - which makes it easier for extensions regarding the output and still being able to switch the Tester-class without storing output format relevant data in the Tester-class
```

- experiment/PairedTTester.html (1.30)

2005-05-13 02:53 trigg

```
Removed unused import.
```

- core/Utils.html (1.47)

2005-05-09 01:55 fracpete

```
modified the convertToRelativePath method to make it work running under cygwin (cygwin doesn't like upper case drive letters!)
```

- gui/experiment/DatasetListPanel.html (1.15)

2005-05-04 01:23 fracpete

```
Plot2D no longer PrintablePanel, therefore made internal class PlotPanel a PrintablePanel - MouseEvents don't disappear anymore
```

- gui/visualize/VisualizePanel.html (1.23)

2005-05-04 01:22 fracpete

```
no longer PrintablePanel, but again JPanel - MouseEvents in the VisualizePanel disappeared without being consumed in PrintableComponent???
```

- gui/visualize/Plot2D.html (1.22)

2005-04-29 00:06 fracpete

```
uses now weka.gui.LookAndFeel to set the Look'n'Feel - to avoid crashes under Java 1.5
```

- clusterers/forOPTICSAndDBScan/OPTICS\_GUI/OPTICS\_Visualizer.html (1.2)

2005-04-28 05:16 fracpete

```
prints additional information about the current/installed Look'n'Feels
```

- core/SystemInfo.html (1.2)

2005-04-28 05:11 fracpete

```
branches:  1.1.2;
manages now centrally the LooknFeel of the GUI
Java 1.5 crashes Weka with certain combinations (e.g. default theme, Gnome/Linux)
```

- gui/LookAndFeel.html (1.1)
- gui/LookAndFeel.props (1.1)

2005-04-27 23:59 eibe

```
Visualizing Bayes nets shoulds work properly again. Thanks, Ashraf.
```

- gui/graphvisualizer/GraphVisualizer.html (1.5)

2005-04-27 22:37 eibe

```
Small fix for initialization of ranges. Thanks, Ashraf.
```

- core/EuclideanDistance.html (1.6)

2005-04-27 21:13 eibe

```
Optimizing the number of bins was broken for datasets with negative attribute values.
```

- filters/unsupervised/attribute/Discretize.html (1.7)

2005-04-26 00:41 mhall

```
Separate test set evaluation now works properly.
```

- gui/explorer/ClustererPanel.html (1.47)

2005-04-25 23:29 fracpete

```
added the "Pattern" button,  which enables the user to select attributes that match a certain regular expression
```

- gui/AttributeSelectionPanel.html (1.7)

2005-04-21 19:58 fracpete

```
fixed nullpointer exception (caused by getOptions()) if "useResampling" was set to true
```

- classifiers/meta/AdaBoostM1.html (1.25)

2005-04-21 19:57 fracpete

```
made it 1.5 compatible: changed "enum" to "enm"
```

- classifiers/bayes/net/search/local/LAGDHillClimber.html (1.2)

2005-04-21 02:46 cvs\_rrb

```
Fixed parameter handling
```

- classifiers/bayes/net/search/local/RepeatedHillClimber.html (1.3)

2005-04-21 02:46 cvs\_rrb

```
Fixed parameter handling
```

- classifiers/bayes/net/search/global/RepeatedHillClimber.html (1.3)

2005-04-20 05:09 eibe

```
New code for finding the kth-smallest element for a numeric attribute/numeric array. Can be used to find the median. Also re-wrote quicksort. (It now shares most of the code with the median-finding method.) New implementation of quicksort has about the same speed as the old one. Played around for some time with more sophisticated versions of quicksort in Instances but couldn't get improvement in speed that warranted additional complexity.
```

- core/Instances.html (1.60)

2005-04-20 03:52 fracpete

```
added accelerator keys ("mnemonics") and default button
```

- gui/DatabaseConnectionDialog.html (1.3)
- gui/ListSelectorDialog.html (1.5)

2005-04-20 03:47 fracpete

```
double click on a classifier has the same functionality as the edit button (makes the editing faster)
```

- gui/experiment/AlgorithmListPanel.html (1.12)

2005-04-20 03:46 fracpete

```
added accelerator keys ("mnemonics") and default button for keyboard-friendly handling
```

- gui/experiment/RunPanel.html (1.19)
- gui/experiment/SimpleSetupPanel.html (1.9)

2005-04-20 03:46 fracpete

```
added accelerator keys ("mnemonics") and default button for keyboard-friendly handling
```

- gui/experiment/SetupModePanel.html (1.3)
- gui/experiment/SetupPanel.html (1.27)

2005-04-20 03:43 fracpete

```
implements now the Tester-Interface
removed all ouput generation, which is now handled by ResultMatrix-classes
```

- experiment/PairedCorrectedTTester.html (1.7)

2005-04-04 04:13 eibe

```
More info on memory allocation thanks to Peter.
```

- gui/LogPanel.html (1.15)

2005-03-23 22:17 eibe

```
New Reorder filter. Improvement of docs for Copy filter. (Another commit try after file permissions in CVS have been fixed.
```

- filters/unsupervised/attribute/Copy.html (1.2)
- filters/unsupervised/attribute/Reorder.html (1.1)

2005-03-23 22:05 eibe

```
Improvements to ArffViewer from Peter.
```

- gui/arffviewer/ArffPanel.html (1.2)
- gui/arffviewer/ArffTableSorter.html (1.2)

2005-03-23 21:55 eibe

```
New Reorder filter. Improvement of docs for Copy filter. Improvements to ArffViewer. Bug fix in NearestNeighbourSearch option setting. All from Peter.
```

- core/NearestNeighbourSearch.html (1.2)

2005-03-17 22:37 mhall

```
KnowledgeFlow now has a more natural ordering of its toolbars
```

- gui/beans/Beans.props (1.11)
